# Mechanical Stress and Protective Mechanisms in Podocytes: Insights into Hypertensive Nephropathy

**DOI:** 10.3390/ijms26199316

**Published:** 2025-09-24

**Authors:** Si-Jia Du, Wei Huang, Yu Hao, Chun Zhang, Fang-Fang He

**Affiliations:** Department of Nephrology, Union Hospital, Tongji Medical College, Huazhong University of Science and Technology, Wuhan 430022, China; dusijia_327@163.com (S.-J.D.); hw15861341084@163.com (W.H.); timely_rain0504@163.com (Y.H.); drzhangchun@hust.edu.cn (C.Z.)

**Keywords:** podocyte, mechanical force, hypertensive nephropathy, shear stress, Piezo1

## Abstract

Mechanical stress on the glomerular filtration barrier (GFB) exposes podocytes to hydrostatic pressure. Their mechanosensitivity is established, yet the underlying mechanotransduction pathways and responses under hypertension remain unclear. This review examines the mechanical stresses experienced by podocytes in both physiologic and hypertensive conditions and updates the latest extracorporeal techniques used to simulate these forces. Additionally, this review discusses how podocytes respond to these mechanical forces and elucidates the detailed molecular mechanisms involved. Furthermore, we summarize potential protective mechanisms that enable podocytes to withstand mechanical challenges and propose novel therapeutic strategies to delay the progression of hypertensive nephropathy. This review uniquely underscores the importance of biomechanical factors in disease progression and integrates emerging therapeutic strategies targeting podocyte mechanotransduction, offering a novel biomechanical framework for delaying hypertensive nephropathy progression.

## 1. Introduction

Hypertensive nephropathy, one of the leading causes of chronic kidney disease (CKD) and end-stage renal disease (ESRD), imposes a substantial global health burden due to its systemic cardiovascular complications and progressive renal dysfunction [1]. Globally, hypertension affects over 1.4 billion individuals, with approximately 30–40% progressing to proteinuric kidney injury [2]. Central to this pathological progression are podocytes, terminally differentiated epithelial cells that form the final filtration barrier of the glomerulus, whose mechanosensitive properties make them susceptible to hypertensive mechanical forces.

Under physiological conditions, podocytes dynamically adapt to tensile and shear stress through coordinated cytoskeletal remodeling mediated by RhoA/ROCK signaling and focal adhesion kinase (FAK) activation. However, sustained glomerular hypertension disrupts this homeostatic balance, generating pathological biomechanical forces that exceed the compensatory capacity of podocytes. This mechanical overload induces maladaptive responses, such as foot process effacement, pseudocyst formation in subpodocyte spaces, and ultimately podocyte detachment. These events are strongly correlated with progressive albuminuria and glomerulosclerosis.

Emerging evidence underscores the pivotal role of mechanotransduction pathways in this process. Hypertensive mechanical stress induces Piezo1-mediated calcium influx and TRPC6-dependent signaling cascades while concurrently downregulating slit diaphragm components, such as nephrin and podocin. These molecular alterations collectively destabilize the actin cytoskeleton, stimulate profibrotic cytokine release, and activate apoptotic pathways. Consequently, the resultant vicious cycle of podocyte loss and compensatory hypertrophy leads to irreversible glomerular barrier dysfunction. In this review, the protective mechanisms of podocytes involve both intrinsic adaptive responses to mechanical stress, including cytoskeletal regulation and adaptive mechanotransduction signaling, as well as external pharmacological interventions designed to mitigate mechanical stress on podocytes and preserve their structural and functional integrity.

Recent technological advancements in renal mechanobiology, particularly glomerulus-on-a-chip (GoC) systems and elastic resonator interference stress microscopy (ERISM), now enable the precise quantification of podocyte mechanoresponses. These platforms have elucidated critical pressure thresholds beyond which podocytes sustain irreversible structural damage, thereby providing quantitative frameworks for therapeutic intervention. Concurrently, multi-omics analyses have identified zyxin-mediated focal adhesion remodeling and KLF15-dependent transcriptional regulation as key adaptive mechanisms against mechanical stress.

This review synthesizes the current understanding of podocyte mechanobiology in hypertensive nephropathy, examining the mechanical forces acting on podocytes under physiological and hypertensive conditions, the molecular mechanisms underlying mechanotransduction pathways, emerging diagnostic techniques for quantifying glomerular biomechanics, and therapeutic strategies targeting these mechanisms. By integrating clinical trial data, we propose a medical framework based on biomechanical modulation for protecting podocyte integrity and delaying disease progression.

## 2. The Mechanical Forces Endured by Podocytes Under Physiological Conditions

Podocytes are terminally differentiated epithelial cells, with their primary and secondary processes extending to envelop the glomerular basement membrane (GBM) of the capillaries within the glomeruli. Podocytes possess primary, secondary, and tertiary foot processes, all of which harbor an extensive actin cytoskeleton and interdigitate with the foot processes of neighboring podocytes [3]. This cytoskeletal network is integral to cell–matrix interactions and mechanosensing, enabling podocytes to dynamically adapt to various mechanical stresses.

**Figure 1:**
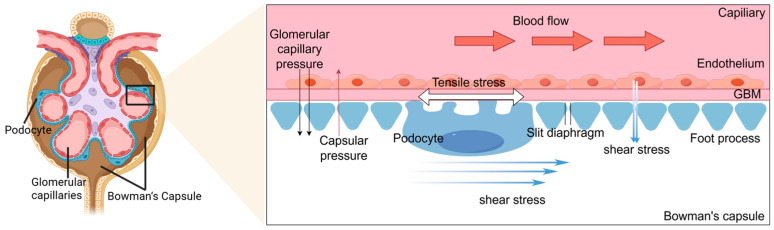
Mechanical forces acting on podocytes. Tensile stresses are generated by blood flow in the capillaries. It includes the pressure difference between the glomerular capillary and Bowman’s capsular pressure, as well as the longitudinal dilation of the glomerular capillaries. The endothelium, the glomerular basement membrane, and podocytes are subjected to tensile stress. The flow of filtrate through the slit diaphragm between the foot process and the flow in the Bowman’s capsule over the surfaces of the podocyte and the cell body both produce shear stresses on podocytes.

Under physiological conditions, podocytes are subjected to mechanical forces originating from both glomerular capillary pressure and glomerular ultrafiltration, including tensile stress and shear stress [4]. These forces are integral to the normal functioning of the glomerulus and are sensed and transduced by the actin cytoskeleton (Figure 1).

### 2.1. The Tensile Stress

The glomerular filtration barrier, which is composed of endothelial cells, the GBM, and podocytes, is subjected to tensile stress. The magnitude of tensile force is influenced by the pressure difference between the glomerular capillary lumen and Bowman’s space, as well as the mechanical properties of the GBM. Under physiological conditions, the GBM provides significant elastic counterforces to balance the increased transmural pressures. Tensile stress is exerted on the capillary wall by blood flow in the capillaries, including circumferential wall stress and axial forces [5]. Circumferential wall stress (hoop stress) arises from the hydraulic pressure difference between the glomerular capillaries and Bowman’s space, driving tubular fluid flow. Axial wall stress originates from the longitudinal distension of glomerular capillaries. Collectively, these stresses act on the glomerular structure, preserving its normal physiological function while influencing the integrity of the filtration barrier.

### 2.2. Shear Stress

Podocytes are subjected to two primary types of shear stress: filtrate flow through the filtration slits between foot processes and the flow in Bowman’s capsule across the surface of primary processes and cell bodies. The shear stress generated by filtrate flow through the filtration slits has been estimated to be approximately 8 Pa [5]. This high level of shear stress arises from the narrow width of the filtration slits (approximately 30–40 nm) and the rapid flow rate of the filtrate. In contrast, the shear stress experienced by podocytes within Bowman’s space is significantly lower, estimated at around 0.05 Pa [5]. This marked difference in the magnitude of shear force highlights that the shear forces acting on podocyte foot processes are a major contributor to podocyte detachment.

## 3. The Response of Podocytes to Mechanical Forces Under Hypertensive Conditions

During aging, some podocytes are lost naturally, but accelerated podocyte loss is a hallmark of CKD. Podocyte damage is now recognized as a causal factor in the progression of hypertensive nephropathy [6]. Podocytes are anchored to the basement membrane solely through their cell processes, and filtrate passes through the subpodocyte space beneath their cell bodies, thereby exposing them to the risk of detachment [7]. Under chronic pathological conditions, such as glomerular hypertension or hyperfiltration, the capacity of podocytes to counterbalance increased biomechanical forces becomes limited, resulting in cellular stress, detachment, and ultimately glomerular dysfunction [8].

Foot process effacement is characterized by the flattening and broadening of these foot processes [9]. In studies conducted on isolated rat kidneys, it was demonstrated that the pericapillary GBM area can increase by more than 50% when perfused at a pressure of 105 mmHg for 100 min. Notably, while both podocyte foot processes and the slit diaphragm between them lengthened under high perfusion pressures, their widths remained unchanged compared to lower perfusion pressures [10,11]. Microscopic evaluations utilizing both scanning and transmission electron microscopy revealed that the structural integrity of podocyte foot processes is maintained even under these pressure challenges. However, when pressure exceeds what podocytes can tolerate, a signaling cascade is activated that prompts closure of the slit by rearranging the actin cytoskeleton. Consequently, foot processes retract and broaden—a phenomenon referred to as foot process effacement [12]. In most instances, these alterations are reversible, thus allowing podocytes to revert to their normal structure once mechanical stressors are alleviated.

Nevertheless, if elevated mechanical forces persist over time, structural derangements may become irreversible, leading to potential detachment from the GBM. Following an initial loss of podocytes, remaining cells often respond with compensatory hypertrophy in order to maintain complete coverage of the GBM. However, continued loss of podocytes accumulates over time; upon reaching a critical threshold (approximately 40%), remaining podocytes lose their capacity for further compensatory hypertrophy in response to cell loss [7]. Chronic mechanical stress or inadequate adaptation results in the formation of pseudocysts within subpodocyte spaces, further compromising adhesion stability [7]. The development of pseudocysts signifies a failure of podocytes to maintain normal filtrate drainage, which contributes to podocyte detachment.

## 4. Extracorporeal Techniques for Simulating Mechanical Forces

Innovative extracorporeal techniques, including GoC microfluidic systems, ERISM, and rotational force devices, now enable precise replication and quantification of biomechanical stresses on podocytes under physiologically controlled conditions (Table 1). These methodological advances have yielded critical insights into how pathological mechanical forces drive podocyte injury and glomerular filtration barrier dysfunction in hypertensive nephropathy.

### 4.1. GoC

GoC systems utilize advanced microfluidic technologies to replicate in vivo mechanical forces critical for renal filtration physiology. These devices typically feature a bilayer structure with a porous membrane (pore size of 3 μm) or a three-dimensional hydrogel matrix that separates the endothelial and podocyte layers, thereby enabling basal-pedunculated fluid transport and inducing physiologic deformation of the GBM [13]. These microfluidic devices are specifically engineered to recreate the hemodynamic microenvironment inherent to renal filtration units by precisely controlling fluid dynamics and mechanical stress. Its advantages include higher physiological relevance compared to static culture, the ability to construct personalized disease models based on patient-specific induced pluripotent stem cells [14] while avoiding species-specific limitations inherent in animal studies, and compatibility with traditional testing methods. Its limitations include subphysiological pressure ranges (max 0.50 kPa vs. in vivo 1.3–3.0 kPa [15]) due to the absent GBM and challenges in maintaining differentiated podocytes in the long term [16].

Current applications are focused on modeling hyperfiltration-induced podocyte injury, studying altered drug sensitivity (e.g., enhanced doxorubicin toxicity under mechanical stress), and addressing inherited glomerular diseases (e.g., Alport syndrome) through patient-specific cell integration [15]. Such systems facilitate real-time assessments of selective permeability while allowing for molecular assays related to mechanical permeability. The GoC platform achieves unprecedented accuracy in kidney disease modeling and drug testing through its ability to simulate the in vivo biomechanical microenvironment.

### 4.2. ERISM

ERISM is an innovative microscopy technique designed to monitor mechanical forces between cells and substrates with exceptional precision [17]. ERISM employs elastic microcavities to detect cell-induced substrate deformations through interferometric methods, achieving remarkable sensitivity and a broad dynamic range. This technique facilitates the long-term monitoring of cellular forces without causing phototoxicity or necessitating cell detachment, making it particularly well-suited for investigating the chronic effects of mechanical stress on podocytes.

In comparison to conventional atomic force microscopy, ERISM offers several advantages, including non-contact measurements, a wide-band dynamic response, and the capability to detect simultaneous multiaxial stresses at a subcellular scale. This technique has been successfully applied in studies of cellular mechanics, such as quantitatively monitoring the cytoskeletal reorganization process in endothelial cells subjected to fluid shear stress. Recent research has further integrated ERISM with microfluidic chips, enabling long-term (>72 h) in situ observation of cell–substrate interaction mechanics under dynamic culture conditions (e.g., cyclic tensile strain ranging from 10% to 20%). These advancements open new avenues for exploring mechanical feedback mechanisms within organ-on-chip systems [17].

### 4.3. Rotational Force Device

The Rotational Force Driven Pressure System represents an innovative in vitro technology designed to replicate mechanical forces pertinent to hypertensive kidney disease research. This centrifugal force-based apparatus comprises an aluminum spindle, a Teflon mounting base for cell culture plates, and a programmable brushless DC motor that generates unidirectional pressure through rotational force. The system is capable of applying physiologically relevant hydrostatic pressure (ranging from 0.4 to 13.4 mmHg) by modulating the rotational speed [18].

A key design advantage of this system lies in its precise control over pressure, achieved through the regulation of rotational speed via a microcontroller. This mechanical model more accurately reproduces the pathophysiology associated with hemodynamic pressures without necessitating direct pharmacological intervention, unlike traditional chemical induction methods, such as angiotensin II therapy. Consequently, this technology addresses a significant gap in hypertension research, facilitating physiologically relevant mechanistic and molecular investigations.

## 5. The Key Molecules in Mechanical Signal Transduction Within Podocytes

**Figure 2:**
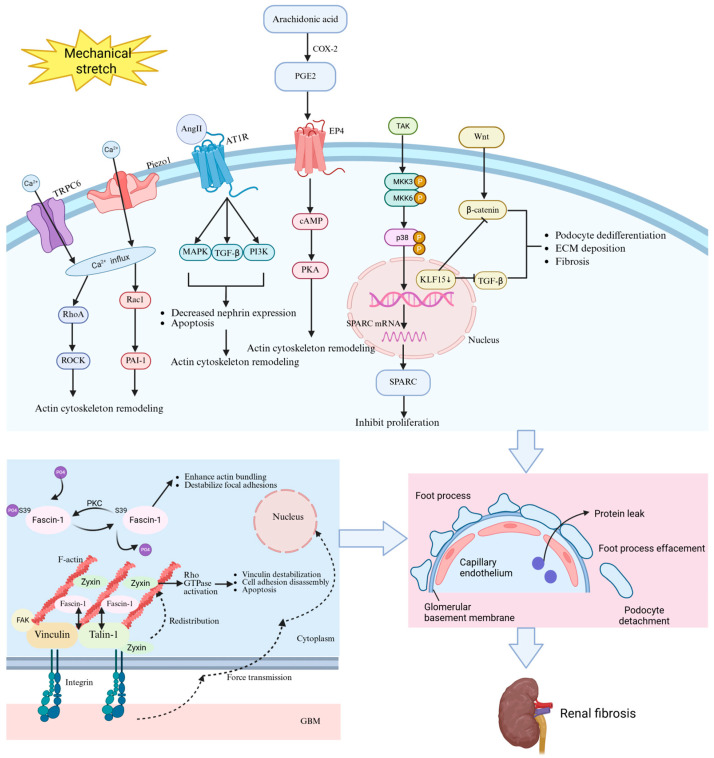
Mechanism of podocyte injury induced by mechanical stretch. Mechanical stress activates Piezo1 and TRPC6 channels, leading to Ca^2+^ influx and subsequent remodeling of the F-actin cytoskeleton. Concurrently, mechanical stretch stimulates arachidonic acid metabolism via COX-2, generating PGE2, which binds to the EP4 receptor, activating the PKA signaling pathway and thereby disrupting the actin cytoskeleton. Ang II activates AT1R, thereby initiating the MAPK, TGF-β, and PI3K signaling cascades, ultimately resulting in the downregulation of nephrin, induction of apoptosis, and reorganization of the cytoskeleton. The downregulation of KLF15 leads to the alleviation of inhibition on both the Wnt/β-catenin and TGF-β signaling pathways, resulting in the activation of the fibrotic pathway. Mechanical stress leads to increased SPARC expression, which can inhibit podocyte proliferation and facilitate ECM deposition. Mechanical stress also enhances actin polymerization through fascin-1 dephosphorylation while simultaneously disrupting focal adhesions, thereby compromising cell–matrix adhesion. The translocation of zyxin to actin-rich regions, along with the destabilization of integrin-associated proteins talin-1 and vinculin, accelerates focal adhesion disassembly. These molecular changes collectively result in impaired integrity of the glomerular filtration barrier, characterized by protein leakage, foot process effacement, and podocyte detachment. Overall, these pathophysiological alterations contribute to the progression of renal fibrosis. TRPC6: transient receptor potential canonical 6; PGE2: prostaglandin E2; COX-2: cyclooxygenase-2; EP4: E-prostanoid receptor 4; PKA: protein kinase A; Ang II: angiotensin II; AT1R: angiotensin II type 1 receptor; MAPK: mitogen-activated protein kinase; TGF-β: transforming growth factor-β; PI3K: phosphatidylinositol 3-kinase; KLF15: Krüppel-like Factor 15; SPARC: secreted protein acidic and rich in cysteine; ECM: extracellular matrix. The solid arrow denotes promotion; the dashed arrow represents the direction of force transmission or zyxin translocation; the bidirectional arrow indicates interaction; and the T-shaped arrow signifies inhibition.

Podocyte responses to mechanical forces in hypertensive nephropathy are orchestrated by a complex network of mechanosensitive molecules and signaling pathways. Ion channels, including Piezo1 and transient receptor potential channel type 6 (TRPC6), play a role in converting mechanical forces into biochemical signals within podocytes. Key molecules, such as angiotensin II (Ang II), zyxin, fascin-1, prostaglandin E2 (PGE2), secreted protein acidic and rich in cysteine (SPARC), and Krüppel-like Factor 15 (KLF15), also participate in mechanical transduction. Under mechanical stretch, dysregulation of these molecules disrupts calcium homeostasis, cytoskeletal integrity, focal adhesion dynamics, and transcriptional programs, ultimately driving foot process effacement, detachment, and glomerulosclerosis (Table 2). Understanding these mechanotransduction mechanisms provides the critical molecular foundation for developing targeted interventions (Figure 2).

### 5.1. Piezo1

Piezo1, a mechanosensitive and nonselective cation channel, is extensively expressed in the glomeruli, particularly within podocytes, mesangial cells, and endothelial cells. Piezo1 is activated by alterations in membrane tension induced by mechanical stimuli, including shear stress, stretch, compression, and osmotic stress. Upon activation, Piezo1 exhibits permeability to Na^+^, K^+^, Ca^2+^, and Mg^2+^, demonstrating a slight preference for calcium ions [19]. In podocytes, Piezo1 is localized at the cell membrane and plays a crucial role in sensing mechanical forces. It functions as a mechanosensor that converts mechanical stimuli into intracellular signals [20]. Under hypertensive conditions, Piezo1 demonstrates increased expression in both podocytes and mesangial cells, as evidenced by RNAscope in situ hybridization and single-cell RNA sequencing [21].

Activation of Piezo1 through mechanical stretch or pharmacological agonists (e.g., Yoda1) induces remodeling of the F-actin cytoskeleton via the RhoA/Rac1 GTPase signaling pathways. This structural reorganization results in nuclear envelope deformation, DNA double-strand breaks, and mitotic catastrophe within podocytes—manifested by γH2AX foci accumulation and aberrant nuclear morphology [22]. In hypertensive models, glomerular hypertension amplifies Piezo1-mediated mechanotransduction, resulting in the upregulation of injurious markers (e.g., PAI-1, SGK1) along with foot process effacement [23].

Importantly, inhibition of Piezo1 through GsMTx4 or genetic knockout alleviates these pathological changes, highlighting its pivotal role in mechanotransduction [23]. In addition, Rac inhibitors have also been shown to reduce the expression of damage markers [24]; this highlights the downstream effector function of Rac1 within Piezo1-mediated signaling pathways. Collectively, Piezo1 acts as a key sensor for mechanical stress in podocytes, translating hemodynamic overload into molecular cascades that contribute to the glomerular dysfunction observed in hypertensive nephropathy.

### 5.2. TRPC6

TRPC6, a nonselective cation channel belonging to the TRP superfamily, has been implicated in glomerular mechanotransduction, particularly under hypertensive conditions. Predominantly localized at the slit diaphragm of podocytes, TRPC6 interacts with key structural proteins, such as podocin and nephrin, thereby forming a mechanosensitive signaling complex [25]. This specific spatial distribution enables TRPC6 to respond effectively to the mechanical forces generated by glomerular capillary hypertension, which is a hallmark of hypertensive nephropathy.

TRPC6 functions as a stretch-activated channel or serves as a downstream effector within mechanical signaling pathways. The activation of TRPC6 under mechanical stress involves phospholipase C (PLC)-dependent mechanisms [26]; elevated levels of diacylglycerol (DAG) resulting from Gq/11-coupled receptor activation facilitate channel opening [27]. Consequently, this results in an influx of Ca^2+^ that triggers cytoskeletal reorganization and RhoA/ROCK-mediated signaling pathways [28], exacerbating podocyte injury through actin dysregulation and foot process effacement. In hypertensive states, there is an upregulation in both the expression and activity of TRPC6. Genetic mutations or hyperactivation of TRPC6 can disrupt calcium homeostasis, amplifying mechanotransduction signals that contribute to podocyte detachment and proteinuria. Furthermore, TRPC6 exhibits synergistic interactions with purinergic P2X4 channels that mediate ATP-dependent mechanical responses [27], suggesting a complex interplay between chemical and mechanical stimuli in podocyte injury.

### 5.3. Ang II and AT1R

Ang II, a potent vasoactive peptide of the renin–angiotensin–aldosterone system (RAAS), exerts its effects primarily through the type 1 receptor (AT1R), a G protein-coupled receptor localized on podocytes [29]. Under physiological conditions, Ang II-AT1R signaling regulates glomerular hemodynamics and podocyte contractility. However, in hypertensive nephropathy, mechanical stress induced by glomerular hypertension disrupts this balance, triggering maladaptive molecular cascades. Mechanical stretch of podocytes can activate the local RAAS, leading to increased de novo synthesis of Ang II [30]. This upregulation occurs via mechanotransduction pathways involving integrins, FAK, and Rho GTPases, which stimulate angiotensinogen expression and subsequent Ang II production. Elevated levels of Ang II bind to AT1R on podocytes, activating downstream signaling cascades, including the mitogen-activated protein kinase (MAPK)/extracellular regulated protein kinase (ERK) pathway, phosphatidylinositol 3-kinase (PI3K)/Akt pathway, and transforming growth factor-β (TGF-β)/Smad pathways [31]. These interconnected pathways converge to induce podocyte injury characterized by downregulation of nephrin expression, remodeling of the actin cytoskeleton, and apoptosis.

In hypertension, nephrin expression is significantly reduced due to repression mediated by Ang II-AT1R interactions that affect both transcriptional regulation and post-translational stability. Furthermore, Ang II-AT1R signaling promotes oxidative stress as well as endoplasmic reticulum (ER) stress—factors that further compromise nephrin stability. Concurrently, overexpression of AT1R amplifies Ang II-induced podocyte injury, creating a feed-forward loop that exacerbates proteinuria and glomerulosclerosis.

Notably, pharmacological inhibition of AT1R or enhancement in nephrin expression via PPAR-γ agonists has been shown to mitigate these detrimental effects [32]; this underscores the therapeutic potential inherent in targeting this axis within hypertensive nephropathy.

### 5.4. Zyxin

Zyxin is a crucial protein associated with focal adhesion and actin dynamics. Under physiological conditions, zyxin displays a polarized distribution on the ventral surfaces of podocytes. It concentrates at focal adhesions and along actin stress fibers. Zyxin plays a significant role in facilitating the assembly of actin filaments and stabilizing these stress fibers, thereby enabling effective force transmission from the extracellular matrix (ECM) to the nucleus.

Functionally, zyxin acts as both a mechanotransducer and a scaffold protein, exerting its effects through direct interactions with various actin-binding proteins, such as vinculin and members of the Ena/VASP family. These interactions not only stabilize actin stress fibers but also promote the elongation of actin filaments. Mechanical stretch triggers the translocation of zyxin from focal adhesions to both actin filaments and the actin-rich center (ARC) [33], orchestrating actin polymerization and cell adhesion processes.

Mechanistically, zyxin facilitates force transmission from integrins to the actin cytoskeleton by recruiting Ena/VASP proteins to focal adhesions, which promotes increased actin polymerization via the inhibition of barbed-end capping mediated by its EVH1 domain [34]. In podocytes subjected to mechanical stretch, zyxin accumulates at ARCs in a LIM domain-dependent manner, where it synergizes with Rho GTPase signaling pathways (e.g., Rac1/Cdc42) to modulate actin dynamics while maintaining integrity in podocyte foot processes.

Notably, a deficiency in zyxin disrupts this mechanotransductive axis, leading to reduced expression levels of vinculin/VASP, impaired remodeling of the actin cytoskeleton, and compromised stability at focal adhesions.

In hypertensive models (e.g., DOCA-salt-treated mice) and human diabetic nephropathy, zyxin is significantly upregulated in podocytes, particularly in glomerular regions that experience heightened mechanical stress. This upregulation correlates strongly with effacement of podocyte foot processes and subsequent development of proteinuria [33].

Chronic hyperactivation of zyxin-mediated pathways may paradoxically disrupt actin homeostasis, leading to podocyte detachment and dysfunction of the glomerular barrier. Single-cell transcriptomics further demonstrate that zyxin expression is significantly enriched in podocytes compared to other renal cell types, highlighting its specificity in mediating hypertensive injury.

### 5.5. Fascin-1

Fascin-1 is a conserved actin-bundling protein that plays a crucial role in podocytes. Within these cells, fascin-1 colocalizes with nephrin in podocytes and aligns along actin stress fibers and filopodia under both unstretched and mechanically stretched conditions. Structured illumination microscopy has demonstrated its enrichment in the ARC of podocytes, where radial stress fibers converge. As a 55 kDa actin-binding protein, fascin-1 possesses tandem actin-binding domains and undergoes post-translational regulation through phosphorylation at serine 39 (Ser-39), a modification mediated by protein kinase C (PKC) activity [35]. Fascin-1 exerts its mechanoprotective function through actin bundling and modulation of focal adhesions. Phosphorylation of Ser-39 suppresses fascin-1’s bundling activity, resulting in retraction of filopodia and reduced cell adhesion. Conversely, dephosphorylation at this residue enhances actin bundling, promoting podocyte spreading and stabilization under mechanical stretch. Additionally, fascin-1 interacts with vinculin and talin-1 at focal adhesions, effectively bridging the actin cytoskeleton to integrins that mediate ECM adhesion [36]. This interaction is critical for force transmission and mechanosensing.

In hypertensive nephropathy, sustained glomerular hypertension imposes chronic mechanical strain on podocytes, triggering a compensatory yet maladaptive response in fascin-1 dynamics. Mechanical stretch induces rapid dephosphorylation of Ser-39, transitioning fascin-1 from an inactive (phosphorylated) state to an active (dephosphorylated) conformation. This post-translational modification enhances actin bundling but compromises focal adhesion integrity; this is evidenced by reduced vinculin recruitment and diminished capacity for podocyte adhesion. While total expression of fascin-1 remains unchanged, the loss of Ser-39 phosphorylation disrupts remodeling of the actin cytoskeleton, leading to simplification of foot processes and detachment from the GBM.

Increased expression of a phosphomimetic mutant (S39D) exacerbates detachment induced by mechanical stress, whereas non-phosphorylatable S39A mutants partially maintain podocyte integrity. This positions fascin-1 as a molecular switch that balances actin bundling and adhesion dynamics in response to hemodynamic forces.

### 5.6. PGE2

PGE2 is a bioactive lipid mediator produced by the catalysis of cyclooxygenase-2 (COX-2) from arachidonic acid [37]. Podocytes express both COX-1 and COX-2; however, COX-2 expression is significantly upregulated in response to mechanical stress, such as fluid flow shear stress (FFSS). PGE2 exerts its biological effects through G-protein-coupled E-prostanoid receptors (EPs), particularly EP2 and EP4. These receptors, EP2 and EP4, are prominently localized in podocytes and dynamically regulated by hemodynamic stress, with EP4 exhibiting constitutive expression and EP2 showing inducible upregulation under stressful conditions [38].

Hypertensive conditions impose increased FFSS and glomerular hypertension, leading to sustained COX-2 induction and PGE2 overproduction in podocytes. PGE2 activates the cAMP/PKA pathway via the EP4 receptor, disrupting the balance of AKT/GSK-3β signaling and inhibiting AKT phosphorylation [39]. This disruption adversely affects actin cytoskeleton organization and podocyte adhesion. The synergistic activation of the p38 MAPK and ERK1/2 pathways by EP2/EP4 promotes pro-inflammatory gene expression as well as ECM remodeling. Furthermore, the oxidative stress present in hypertensive environments collaborates with PGE2 to enhance MAPK activation [40], creating a feed-forward loop that exacerbates podocyte injury.

Importantly, mechanical strain also activates the epidermal growth factor receptor (EGFR) [41], which potentiates ERK1/2 and c-Jun N-terminal kinase (JNK) signaling independently of EP4, thereby exacerbating podocyte hypertrophy and detachment. The mechanical stress-induced expressions of COX-2 and EP4 may amplify the effects of PGE2 through an autocrine/paracrine mechanism [41], further contributing to podocyte damage.

### 5.7. SPARC

SPARC, a matricellular glycoprotein, exerts pleiotropic effects mediating cell–matrix interactions. As a member of the matricellular protein family, SPARC is constitutively expressed in podocytes under physiological conditions and becomes activated in response to immune-mediated podocyte injury. Immunohistochemical analyses and in situ hybridization studies reveal that SPARC is predominantly localized within the cytoplasm of podocytes. Its expression and secretion into the ECM are markedly enhanced under mechanical strain, with this increase being transcriptionally regulated.

Mechanical stretch-induced overexpression of SPARC in podocytes occurs through activation of the p38 MAPK pathway [42]. Cyclical mechanical strain triggers phosphorylation of p38, which subsequently stimulates transcriptional upregulation of both SPARC mRNA and protein levels. Furthermore, the renin–angiotensin system, which is activated during hypertensive states, also contributes to elevated expression levels of SPARC. The AT1R blocker losartan has been shown to suppress this overexpression, indicating a role for the renin–angiotensin system in regulating SPARC.

Elevated levels of SPARC exacerbate podocyte injury by inhibiting mitogenic growth factors, such as PDGF, vascular endothelial growth factor (VEGF), and bFGF, thereby diminishing proliferative capacity across various tissue systems. Furthermore, it can directly bind to and modify matrix proteins, disrupting focal cellular adhesions while promoting apoptosis via TGF-β1 activation. Moreover, SPARC synergizes with a disintegrin and metalloproteinase with thrombospondin motifs-1 (ADAMTS1) to accelerate ECM remodeling, collagen deposition, and glomerulosclerosis [43].

### 5.8. KLF15

KLF15, a zinc finger transcription factor that belongs to the Sp1/KLF family, is predominantly localized in glomerular podocytes, renal tubular epithelial cells, and mesangial cells. Structurally, KLF15 contains a conserved C-terminal C_2_H_2_ zinc-finger motif that facilitates DNA binding to GC-rich promoter regions of target genes involved in cytoskeletal dynamics, cell adhesion, and ECM remodeling. Functionally, KLF15 acts as a molecular switch regulating podocyte differentiation and stress responses [44].

Under physiological conditions, KLF15 maintains podocyte homeostasis through dual mechanisms: it stabilizes the actin cytoskeleton by upregulating synaptopodin and nephrin expression while simultaneously suppressing profibrotic signaling pathways such as Wnt/β-catenin and TGF-β/Smad3 [45]. In hypertensive nephropathy, mechanotransduction pathways activated by elevated glomerular capillary pressure lead to downregulation of KLF15 expression in podocytes. Mechanical stress triggers the epithelial-to-mesenchymal transition and dedifferentiation of podocytes—processes mediated by reduced levels of KLF15. Specifically, reduced KLF15 expression results in derepression of connective tissue growth factor (CTGF) induced by TGF-β1, along with activation of the Wnt/β-catenin signaling pathways; this promotes ECM deposition (e.g., fibronectin, collagen IV) and contributes to glomerulosclerosis. KLF15 plays a crucial role in stabilizing focal adhesions and actin filaments through modulation of zonula occludens-1 (ZO-1) and synaptopodin. Its loss results in podocyte foot process effacement and subsequent barrier dysfunction. Therapeutic restoration of KLF15 via retinoic acid or pharmacological inhibition of TGF-β/CTGF signaling has been shown to reverse podocyte dysfunction as well as ECM accumulation in preclinical models [18].

Collectively, KLF15 functions as a mechanoresponsive guardian for maintaining podocyte homeostasis. It can disrupt maladaptive mechanotransduction processes and preserve glomerular function in hypertensive nephropathy.

## 6. Treatment for Protecting Podocytes from Mechanical Forces

The traditional methods for protecting podocytes from mechanical damage include angiotensin-converting enzyme inhibitors (ACEi), angiotensin receptor blockers (ARBs), and direct renin inhibitors. These methods have undergone rigorous validation and are widely used in clinical practice, providing substantial benefits to patients. Furthermore, as research into the mechanotransduction signals of podocytes deepens, intervention strategies targeting novel targets (e.g., TRPC6 and Piezo1) are emerging, with the potential to offer safer and more effective treatment options (Table 3).

### 6.1. ARB or ACEi Therapy

Ang II serves as a renal microvascular regulator, facilitating vasoconstriction of both the afferent and efferent arterioles. ACEi and ARBs are effective in managing Ang II-mediated intraglomerular hypertension and mitigating increased tensile stress on podocytes. The injurious effects of Ang II on podocytes arise from various signaling mechanisms, including caveolin 1-mediated downregulation and dephosphorylation of nephrin, disruption of the actin cytoskeleton via the Rho-Rock pathways, and p38 MAPK-mediated apoptotic processes. Additionally, Ang II can elevate intracellular calcium concentrations in podocytes by stimulating TRPC6-mediated release of calcium from intracellular stores [46].

The involvement of RAAS signaling in the regulation of the podocyte structure and homeostasis provides a mechanistic foundation for the current recommendations advocating the use of ACEi and ARBs in treating proteinuric kidney disease.

### 6.2. Direct Renin Inhibitor

Aliskiren, the first orally active Direct Renin Inhibition (DRI), inhibits renin’s catalytic conversion of angiotensinogen to angiotensin I, thereby suppressing the entire RAAS cascade at its origin. This contrasts with ACEi or ARBs, which trigger compensatory rises in plasma renin activity and incomplete RAAS blockade [47].

Prorenin binds to prorenin receptor (PRR) on podocytes, triggering Ang II-independent activation of the ERK/MAPK pathways and promoting cytoskeletal disruption. Aliskiren inhibits both renin and receptor-bound prorenin activity, downregulating PRR expression and interrupting profibrotic signaling (e.g., TGF-β, PAI-1) in podocytes [48].

In diabetic transgenic rats, aliskiren decreased albuminuria, podocyte injury markers, and glomerulosclerosis, correlating with reduced renal Ang II and PRR expression. These benefits occurred independently of blood pressure reduction, highlighting direct podocyte protection. The AVOID trial demonstrated that aliskiren add-on therapy to losartan further reduced albuminuria by 20% in diabetic nephropathy [49]. This suggests synergistic renoprotection potentially mediated by alleviating podocyte stress.

Targeting the initial step of the renin–angiotensin cascade with DRIs offers a mechanistically grounded strategy to mitigate podocyte injury under mechanical stress, complementing conventional RAAS blockade by concurrently suppressing Ang II-dependent and PRR-driven pathways.

### 6.3. TRPC6 Blockade

Targeting mechanosensitive ion channels and signaling hubs represents a novel therapeutic frontier. TRPC6, a calcium-permeable channel activated by mechanical stress, has been implicated in podocyte injury via dysregulated calcium signaling and an imbalance of RhoA-Rac1 GTPase activity [50]. RhoA/ROCK1 activation drives profound cytoskeletal rearrangement and foot process effacement, ultimately compromising the glomerular filtration barrier, manifesting as proteinuria. Pharmacological inhibition of TRPC6 (e.g., SAR-7334, BI-749327, tetrandrine [51]) or genetic ablation attenuates this mechanical stress response. Preliminary clinical evidence suggests that the use of the selective TRPC6 inhibitor BI-749327 significantly reduces proteinuria, alleviates glomerulosclerosis, and decreases tubulointerstitial fibrosis in rats treated with doxorubicin [52]. Preliminary clinical evidence indicates that the natural alkaloid tetrandrine, a nonselective Ca^2+^ channel blocker, suppresses TRPC6-mediated calcium influx in cultured podocytes, reducing calpain-1 activity by 60% and restoring talin-1 and nephrin expression in adriamycin-induced focal segmental glomerulosclerosis (FSGS) rat models. These effects correlate with a 42% reduction in 24-h proteinuria and significant improvements in podocyte foot process effacement (fusion rate decreased from 80–100% to 30–40%) [53]. BI 764198 is currently under evaluation in a phase II, multicenter, randomized, placebo-controlled trial (NCT05213624) in patients with primary or TRPC6-mediated monogenic FSGS. This trial aims to evaluate its efficacy in reducing proteinuria and preserving glomerular function, representing a significant step toward clinical translation [54]. Thus, targeted inhibition of TRPC6 emerges as a promising therapeutic strategy specifically aimed at interrupting the deleterious cycle of mechanical stress-induced calcium dysregulation and cytoskeletal disruption in vulnerable podocytes, offering potential for preserving glomerular function in proteinuric kidney diseases.

### 6.4. Piezo1 Modulation

Piezo1 mediates the inward flow of calcium by sensing mechanical forces. Calcium signaling activates the Rac1 pathway, which enhances actin cytoskeleton remodeling and reduces podocyte migration. Concurrently, TRPC6 synergistically enhances calcium influx, creating a positive feedback loop that leads to podocyte apoptosis. However, Piezo1 deficiency results in nuclear membrane collapse, increased γH2AX markers, and triggers mitotic catastrophe. Pharmacological inhibition of Piezo1 channels through the peptide antagonist GsMTx4 exhibited significant antifibrotic effects across multiple experimental models. In a murine model of unilateral ureteral obstruction, subcutaneous administration of GsMTx4 (10 mg/kg every other day) resulted in a reduction in collagen I and fibronectin protein levels by over 50% at seven days post-surgery. Masson’s trichrome staining further confirmed a marked attenuation of tubulointerstitial fibrosis in treated animals. These therapeutic benefits were also observed in a folic acid-induced nephropathy model, where GsMTx4 effectively normalized Piezo1 overexpression (a 70% decrease, *p* < 0.05) and reduced fibronectin deposition by more than 40% [55]. In a model of hypertensive nephropathy, the Piezo1-specific small molecule inhibitor Dooku1 effectively blocks mechanical stretch-induced Rac1 activation and attenuates the expression of podocyte injury markers [23].

Yoda1, a known agonist of Piezo1, mimics mechanical stretch and induces both cytoskeletal remodeling and apoptosis in podocytes. However, low doses of Yoda1 have been observed to enhance residual podocyte function. Clinical studies indicate that renal biopsies from patients with lupus nephritis demonstrate a positive correlation between Piezo1 expression levels and proteinuria severity. Furthermore, inhibition of Piezo1 has been shown to ameliorate podocyte injury and reduce proteinuria in conditions such as FSGS, diabetic nephropathy, and lupus nephritis [56]. The therapeutic targeting of Piezo1 presents novel strategies for addressing nephropathies related to mechanical stress on podocytes. However, the pharmacological targeting of Piezo1 is not without challenges. As a widely expressed mechanosensor, systemic inhibition of Piezo1 may lead to off-target effects, including impaired vascular development, erythrocyte volume dysregulation, and compromised immune responses. Therefore, developing podocyte-specific delivery systems or allosteric modulators that fine-tune Piezo1 activity without complete inhibition may be necessary to minimize adverse effects [57].

### 6.5. Combination Therapies

A highly promising future direction lies in combination therapy. RAAS inhibitors can reduce glomerular capillary pressure, whereas TRPC6 or Piezo1 inhibitors can directly block pathological calcium influx and the subsequent downstream signaling within podocytes. Preclinical studies have demonstrated that the combination of GsMTx4 and losartan reduced proteinuria by 42% in doxorubicin-induced nephropathy rats, significantly outperforming monotherapy, which achieved a reduction of 28%. Pharmacodynamic data indicate that BI 764198 exhibits linear pharmacokinetic characteristics within the dose range of 20–160 mg and can be flexibly combined with RAAS inhibitors. Phase I trial data for the TRPC6 inhibitor BI 764198 confirmed no significant interaction with CYP3A4 substrates, such as midazolam, suggesting that its combination with RAAS inhibitors metabolized by CYP3A4 does not necessitate dose adjustments [58].

In conclusion, the therapeutic options for podocyte protection are expanding beyond RAAS inhibitors. Although the development of Piezo1 inhibitors faces considerable challenges in terms of selectivity and drug-like properties, the diversity of identified chemotypes offers a solid foundation for future optimization. Strategically combining mechanosensitive channel inhibitors with established RAAS blockers represents a rational and promising multi-target approach to more effectively halt the progression of hypertensive kidney disease.

## 7. Conclusions and Future Directions

The investigation of mechanical forces acting on podocytes in hypertensive nephropathy has revealed a complex interplay among hemodynamic stress, cellular mechanotransduction, and glomerular homeostasis. This review clarifies that podocytes, functioning as mechanosensitive sentinels at the GFB, undergo adaptive cytoskeletal remodeling in response to physiological tensile and shear stress. However, persistent hypertension imposes maladaptive mechanical overload, leading to foot process effacement, disruption of the slit diaphragm, and detachment of podocytes—pathological events that are strongly correlated with proteinuria and glomerulosclerosis.

Crucial mechanotransduction pathways, including Piezo1-mediated calcium influx, TRPC6 signaling, and zyxin-dependent actin remodeling, emerge as pivotal determinants of podocyte loss. Notably, advancements in extracorporeal simulation technologies, such as GoC models and ERISM, have provided unprecedented insights into the pressure thresholds for podocyte injury. These innovations offer quantitative frameworks for therapeutic intervention.

The distinctive role of hemodynamic forces in hypertension highlights the necessity for targeted therapies that address biomechanical dysregulation. Current clinical interventions, such as RAAS blockade, partially mitigate podocyte stress. Nevertheless, the advent of molecular targeted therapies, such as TRPC6 inhibitors and Piezo1 modulators, holds promise for precision medical approaches aimed at mitigating mechanical stress in podocytes.

Future investigations should prioritize the development of dynamic monitoring tools capable of quantifying podocyte mechanoresponses in real-time, thereby facilitating early detection of maladaptive remodeling. Collaborative efforts across disciplines, including bioengineering, computational biology, and clinical nephrology, are essential to translate mechanobiological discoveries into personalized treatment paradigms that preserve podocyte integrity while delaying the progression of hypertensive nephropathy.

## Figures and Tables

**Table 1:** Extracorporeal techniques used to simulate mechanical forces.

Technique	Principle/Design	Applications	Advantages	Limitations	Refs.
Glomerulus-on-a-Chip	Microfluidic bilayer with porous membrane or hydrogel matrix simulating GFB.	Modeling hyperfiltration, drug nephrotoxicity screening, inherited glomerular diseases (e.g., Alport syndrome).	High physiological relevance; supports patient-specific iPSC models; compatible with traditional assays; avoids species-specific limitations.	Complex fabrication; subphysiological pressure ranges; podocyte differentiation maintenance.	[13,14,15]
Elastic Resonator Interference Stress Microscopy	Elastic microcavities detect cell-induced substrate deformations via interferometry.	Long-term monitoring of cellular forces (e.g., cytoskeletal dynamics under cyclic tensile strain).	Non-contact; high sensitivity; no phototoxicity.	Requires advanced imaging systems; limited to in vitro models.	[16]
Rotational Force Device	Centrifugal force generates hydrostatic pressure via a programmable motor.	Mechanistic studies of hypertensive nephropathy; mimics glomerular hypertension.	Precise pressure control; physiologically relevant mechanical stress.	Limited to unidirectional pressure; not suitable for shear stress modeling.	[17]

GFB: glomerular filtration barrier; iPSC: induced pluripotent stem cell.

**Table 2:** Molecular mechanisms within podocytes under mechanical stretch.

Molecule	Physiological Function	Mechanism in Hypertensive Nephropathy	Pathological Effects on Functional Abnormalities	Refs.
Piezo1	Mechanosensitive Ca^2+^ channel.	Stretch-induced Ca^2+^ influx activates Rac1, causing cytoskeletal remodeling and DNA damage.	Podocyte detachment, mitotic catastrophe, and glomerulosclerosis.	[18,19,20,21,22,23]
TRPC6	Stretch-activated Ca^2+^ channel interacting with nephrin/podocin.	Mechanical stress activates the PLC/DAG pathway, causing Ca^2+^ overload and RhoA/ROCK activation.	Actin cytoskeleton destabilization and foot process effacement.	[24,25,26,27]
Ang II/AT1R	Mediates vasoconstriction and podocyte injury.	Mechanical stress activates local RAAS, increasing Ang II production and MAPK/TGF-β pathways.	Podocyte apoptosis, oxidative stress, and fibrosis.	[28,29,30,31]
Zyxin	Stabilizes actin cytoskeleton and focal adhesions; mechanotransducer.	Redistributes to actin-rich centers under stretch, activating Rho GTPase signaling.	Foot process effacement, apoptosis, and proteinuria.	[32,33]
Fascin-1	Actin-bundling protein regulated by Ser-39 phosphorylation.	Dephosphorylation under stress enhances actin bundling but destabilizes focal adhesions.	Podocyte detachment and filtration barrier disruption.	[34,35]
PGE2	Lipid mediator synthesized via COX-2; regulates cAMP/PKA signaling.	Mechanical strain upregulates COX-2, activating the EP4 receptor and ERK/JNK pathways.	Actin cytoskeleton disruption, inflammation, and ECM remodeling.	[36,37,38,39,40]
SPARC	Matricellular protein promoting ECM remodeling.	Mechanical stretch induces SPARC via p38 MAPK; synergizes with ADAMTS1.	Glomerulosclerosis and podocyte–mesenchymal transition.	[41,42]
KLF15	Transcription factor maintaining podocyte differentiation.	Downregulated under mechanical stress, derepressing the Wnt/β-catenin and TGF-β pathways.	Podocyte dedifferentiation, ECM deposition, and fibrosis.	[17,43,44]

TRPC6: transient receptor potential canonical 6; PLC: phospholipase C; DAG: diacylglycerol; Ang II: angiotensin II; AT1R: angiotensin II type 1 receptor; RAAS: renin–angiotensin–aldosterone system; MAPK: mitogen-activated protein kinase; TGF-β: transforming growth factor-β; Ser-39: serine 39; PGE2: prostaglandin E2; COX-2: cyclooxygenase-2; PKA: protein kinase A; EP4: E-prostanoid receptor 4; ERK: extracellular regulated protein kinase; JNK: c- Jun N-terminal kinase; ECM: extracellular matrix; SPARC: secreted protein acidic and rich in cysteine; KLF15: Krüppel-like Factor 15.

**Table 3:** Therapeutic strategies for podocyte protection.

Treatment Method	Mechanism of Action	Effects	Advantages	Current Clinical Stage	Limitations	Safety Considerations	Refs.
ACEi/ARB	Inhibit Ang II-AT1R signaling; reduce intraglomerular pressure.	Preserve nephrin expression; reduce proteinuria and podocyte apoptosis.	Rigorously validated; widely used; reduces intraglomerular pressure.	Widely approved and used in clinical practice	May cause hyperkalemia, hypotension, or renal impairment in some patients; incomplete RAAS blockade.	Generally safe with monitoring; contraindicated in pregnancy.	[45]
Direct Renin Inhibitor (Aliskiren)	Suppresses the entire RAAS cascade. Reduces prorenin–PRR binding and downstream ERK/MAPK activation.	Attenuates cytoskeletal disruption; reduces albuminuria and glomerulosclerosis.	Suppresses compensatory renin rise seen with ACEi/ARBs; synergistic with ARBs.	Phase 2 completed (e.g., AVOID trial)	Limited long-term efficacy data; potential for hyperkalemia similar to other RAAS inhibitors.	Monitor for hyperkalemia and renal function; avoid in patients with renal artery stenosis.	[46,47,48]
TRPC6 Blockers	Antagonize TRPC6 channel activity.	Stabilize actin dynamics; attenuate proteinuria.	Targeted interruption of mechanical stress cascade; preclinical efficacy in reducing proteinuria/glomerulosclerosis.	Phase 1 (e.g., BI 764198 trial)	Selectivity issues due to TRPC6 expression in other tissues; limited clinical data on efficacy.	Under investigation; may interact with CYP3A4 substrates; monitor for off-target effects.	[49,50,51]
Piezo1 Modulators	Block Piezo1-mediated mechanotransduction.	Reduce Rac1 activation and podocyte injury.	Novel targeting of mechanical sensor; efficacy in multiple nephropathy models.	Preclinical (e.g., GsMTx4 in animal models)	Systemic inhibition may impair vascular development, erythrocyte function, or immune responses; challenge in selectivity.	High risk of off-target effects; requires podocyte-specific delivery systems to minimize adverse impacts.	[22,52]

ACEi: angiotensin-converting enzyme inhibitor; ARB: angiotensin receptor blocker; Ang II: angiotensin II; AT1R: angiotensin II type 1 receptor; RAAS: renin–angiotensin–aldosterone system; PRR: prorenin receptor; ERK: extracellular regulated protein kinase; MAPK: mitogen-activated protein kinase; TRPC6: transient receptor potential canonical 6.

## References

[1] Zhang Y., Arzaghi H., Ma Z., Roye Y., Musah S. (2024). Epigenetics of Hypertensive Nephropathy. Biomedicines.

[2] Mills K.T., Stefanescu A., He J. (2020). The global epidemiology of hypertension. Nat. Rev. Nephrol..

[3] Kopp J.B., Anders H.-J., Susztak K., Podestà M.A., Remuzzi G., Hildebrandt F., Romagnani P. (2020). Podocytopathies. Nat. Rev. Dis. Primers.

[4] Blaine J., Dylewski J. (2020). Regulation of the Actin Cytoskeleton in Podocytes. Cells.

[5] Endlich N., Endlich K. (2012). The Challenge and Response of Podocytes to Glomerular Hypertension. Semin. Nephrol..

[6] Reynolds P.A. (2020). The mechanobiology of kidney podocytes in health and disease. Clin. Sci..

[7] Kriz W., Lemley K.V. (2016). Mechanical challenges to the glomerular filtration barrier: Adaptations and pathway to sclerosis. Pediatr. Nephrol..

[8] Haydak J., Azeloglu E.U. (2024). Role of biophysics and mechanobiology in podocyte physiology. Nat. Rev. Nephrol..

[9] Kriz W., Shirato I., Nagata M., LeHir M., Lemley K.V. (2013). The podocyte’s response to stress: The enigma of foot process effacement. Am. J. Physiol. Physiol..

[10] Kriz W., Hackenthal E., Nobiling R., Sakai T., Elger M., Hähnel B. (1994). A role for podocytes to counteract capillary wall distension. Kidney Int..

[11] Sakai T., Lemley K.V., Hackenthal E., Nagata M., Nobiling R., Kriz W. (1992). Changes in glomerular structure following acute mesangial failure in the isolated perfused kidney. Kidney Int..

[12] Garg P. (2018). A Review of Podocyte Biology. Am. J. Nephrol..

[13] Doi K., Kimura H., Matsunaga Y.T., Fujii T., Nangaku M. (2022). Glomerulus-on-a-Chip: Current Insights and Future Potential Towards Recapitulating Selectively Permeable Filtration Systems. Int. J. Nephrol. Renov. Dis..

[14] Musah S., Mammoto A., Ferrante T.C., Jeanty S.S.F., Hirano-Kobayashi M., Mammoto T., Roberts K., Chung S., Novak R., Ingram M. (2017). Mature induced-pluripotent-stem-cell-derived human podocytes reconstitute kidney glomerular-capillary-wall function on a chip. Nat. Biomed. Eng..

[15] Doi K., Kimura H., Kim S.H., Kaneda S., Wada T., Tanaka T., Shimizu A., Sano T., Chikamori M., Shinohara M. (2023). Enhanced podocyte differentiation and changing drug toxicity sensitivity through pressure-controlled mechanical filtration stress on a glomerulus-on-a-chip. Lab Chip.

[16] Huang W., Chen Y.-Y., He F.-F., Zhang C. (2024). Revolutionizing nephrology research: Expanding horizons with kidney-on-a-chip and beyond. Front. Bioeng. Biotechnol..

[17] Kronenberg N.M., Liehm P., Steude A., Knipper J.A., Borger J.G., Scarcelli G., Franze K., Powis S.J., Gather M.C. (2017). Long-term imaging of cellular forces with high precision by elastic resonator interference stress microscopy. Nat. Cell Biol..

[18] Yu M.-Y., Kim J.E., Lee S., Choi J.W., Kim Y.C., Han S.S., Lee H., Cha R.H., Lee J.P., Lee J.W. (2020). Krüppel-like factor 15 is a key suppressor of podocyte fibrosis under rotational force-driven pressure. Exp. Cell Res..

[19] Xiao B. (2024). Mechanisms of mechanotransduction and physiological roles of PIEZO channels. Nat. Rev. Mol. Cell Biol..

[20] Drobnik M., Smólski J., Grądalski Ł., Niemirka S., Młynarska E., Rysz J., Franczyk B. (2024). Mechanosensitive Cation Channel Piezo1 Is Involved in Renal Fibrosis Induction. Int. J. Mol. Sci..

[21] Yuan X., Zhao X., Wang W., Li C. (2024). Mechanosensing by Piezo1 and its implications in the kidney. Acta Physiol..

[22] Melica M.E., Antonelli G., Semeraro R., La Regina G., Dafichi T., Fantini C., Carangelo G., Comito G., Conte C., Maggi L. (2025). Piezo1, F-actin Remodeling, and Podocyte Survival and Regeneration. J. Am. Soc. Nephrol..

[23] Ogino S., Yoshikawa K., Nagase T., Mikami K., Nagase M. (2023). Roles of the mechanosensitive ion channel Piezo1 in the renal podocyte injury of experimental hypertensive nephropathy. Hypertens. Res..

[24] Shibata S., Nagase M., Yoshida S., Kawarazaki W., Kurihara H., Tanaka H., Miyoshi J., Takai Y., Fujita T. (2008). Modification of mineralocorticoid receptor function by Rac1 GTPase: Implication in proteinuric kidney disease. Nat. Med..

[25] Wang Q., Tian X., Wang Y., Wang Y., Li J., Zhao T., Li P. (2020). Role of Transient Receptor Potential Canonical Channel 6 (TRPC6) in Diabetic Kidney Disease by Regulating Podocyte Actin Cytoskeleton Rearrangement. J. Diabetes Res..

[26] Staruschenko A., Ma R., Palygin O., Dryer S.E. (2023). Ion channels and channelopathies in glomeruli. Physiol. Rev..

[27] Forst A.-L., Olteanu V.S., Mollet G., Wlodkowski T., Schaefer F., Dietrich A., Reiser J., Gudermann T., Mederos y Schnitzler M., Storch U. (2016). Podocyte Purinergic P2X4 Channels Are Mechanotransducers That Mediate Cytoskeletal Disorganization. J. Am. Soc. Nephrol..

[28] Zhang L., Ji T., Wang Q., Meng K., Zhang R., Yang H., Liao C., Ma L., Jiao J. (2017). Calcium-Sensing Receptor Stimulation in Cultured Glomerular Podocytes Induces TRPC6-Dependent Calcium Entry and RhoA Activation. Cell. Physiol. Biochem..

[29] Zhou Q.-Y., Pan J.-Q., Liu W., Jiang Z.-T., Gao F.-Y., Zhao Z.-W., Tang C.-K. (2025). Angiotensin II: A novel biomarker in vascular diseases. Clin. Chim. Acta.

[30] Durvasula R.V., Petermann A.T., Hiromura K., Blonski M., Pippin J., Mundel P., Pichler R., Griffin S., Couser W.G., Shankland S.J. (2004). Activation of a local tissue angiotensin system in podocytes by mechanical strain. Kidney Int..

[31] Durvasula R.V., Shankland S.J. (2006). The renin-angiotensin system in glomerular podocytes: Mediator of glomerulosclerosis and link to hypertensive nephropathy. Curr. Hypertens. Rep..

[32] Miceli I., Burt D., Tarabra E., Camussi G., Perin P.C., Gruden G. (2010). Stretch reduces nephrin expression via an angiotensin II-AT1-dependent mechanism in human podocytes: Effect of rosiglitazone. Am. J. Physiol. Physiol..

[33] Kliewe F., Siegerist F., Hammer E., Al-Hasani J., Amling T.R.J., Hollemann J.Z.E., Schindler M., Drenic V., Simm S., Amann K. (2024). Zyxin is important for the stability and function of podocytes, especially during mechanical stretch. Commun. Biol..

[34] Hirata H., Tatsumi H., Sokabe M. (2008). Mechanical forces facilitate actin polymerization at focal adhesions in a zyxin-dependent manner. J. Cell Sci..

[35] Chung J.M., Sato O., Ikebe R., Lee S., Ikebe M., Jung H.S. (2022). Structural Analysis of Human Fascin-1: Essential Protein for Actin Filaments Bundling. Life.

[36] Kliewe F., Scharf C., Rogge H., Darm K., Lindenmeyer M.T., Amann K., Cohen C.D., Endlich K., Endlich N. (2017). Studying the role of fascin-1 in mechanically stressed podocytes. Sci. Rep..

[37] Nørregaard R., Kwon T.-H., Frøkiær J. (2015). Physiology and pathophysiology of cyclooxygenase-2 and prostaglandin E2 in the kidney. Kidney Res. Clin. Pract..

[38] Mutsaers H.A., Nørregaard R. (2022). Prostaglandin E2 receptors as therapeutic targets in renal fibrosis. Kidney Res. Clin. Pract..

[39] Faour W.H., Thibodeau J.-F., Kennedy C.R. (2010). Mechanical stretch and prostaglandin E2 modulate critical signaling pathways in mouse podocytes. Cell. Signal..

[40] Mangelsen E., Rothe M., Schulz A., Kourpa A., Panáková D., Kreutz R., Bolbrinker J. (2020). Concerted EP2 and EP4 Receptor Signaling Stimulates Autocrine Prostaglandin E2 Activation in Human Podocytes. Cells.

[41] Martineau L.C., McVeigh L.I., Jasmin B.J., Kennedy C.R.J. (2004). p38 MAP kinase mediates mechanically induced COX-2 and PG EP4 receptor expression in podocytes: Implications for the actin cytoskeleton. Am. J. Physiol. Physiol..

[42] Durvasula R.V., Shankland S.J. (2005). Mechanical strain increases SPARC levels in podocytes: Implications for glomerulosclerosis. Am. J. Physiol. Physiol..

[43] Toba H., Ikemoto M.J., Kobara M., Nakata T. (2022). Secreted protein acidic and rich in cysteine (SPARC) and a disintegrin and metalloproteinase with thrombospondin type 1 motif (ADAMTS1) increments by the renin-angiotensin system induce renal fibrosis in deoxycorticosterone acetate-salt hypertensive rats. Eur. J. Pharmacol..

[44] Rane M.J., Zhao Y., Cai L. (2019). Krϋppel-like factors (KLFs) in renal physiology and disease. eBioMedicine.

[45] Wang L., Lin W., Chen J. (2019). Krüppel-like Factor 15: A Potential Therapeutic Target For Kidney Disease. Int. J. Biol. Sci..

[46] Semenikhina M., Fedoriuk M., Stefanenko M., Klemens C.A., Cherezova A., Marshall B., Hall G., Levchenko V., Solanki A.K., Lipschutz J.H. (2023). β-Arrestin pathway activation by selective ATR1 agonism promotes calcium influx in podocytes, leading to glomerular damage. Clin. Sci..

[47] Hollenberg N.K. (2009). Direct renin inhibition and the kidney. Nat. Rev. Nephrol..

[48] Siragy H.M., Carey R.M. (2010). Role of the Intrarenal Renin-Angiotensin-Aldosterone System in Chronic Kidney Disease. Am. J. Nephrol..

[49] Riccioni G. (2011). Aliskiren in the Treatment of Hypertension and Organ Damage. Cardiovasc. Ther..

[50] Jiang L., Ding J., Tsai H., Li L., Feng Q., Miao J., Fan Q. (2011). Over-expressing transient receptor potential cation channel 6 in podocytes induces cytoskeleton rearrangement through increases of intracellular Ca^2+^ and RhoA activation. Exp. Biol. Med..

[51] Mao L., Ding Y., Yu D., Yin J., Yu J., Pragasam V. (2022). Tetrandrine Attenuates Podocyte Injury by Inhibiting TRPC6-Mediated RhoA/ROCK1 Pathway. Anal. Cell. Pathol..

[52] Meliambro K., He J.C., Campbell K.N. (2024). Podocyte-targeted therapies—Progress and future directions. Nat. Rev. Nephrol..

[53] Ding Y., Tang X., Wang Y., Yu D., Zhu C., Yu J. (2021). Tetrandrine alleviates podocyte injury via calcium-dependent calpain-1 signaling blockade. BMC Complement. Med. Ther..

[54] Trachtman H., Kretzler M., Desmond H.E., Choi W., Manuel R.C., Soleymanlou N. (2023). TRPC6 Inhibitor BI 764198 in Focal Segmental Glomerulosclerosis: Phase 2 Study Design. Kidney Int. Rep..

[55] Zhao X., Kong Y., Liang B., Xu J., Lin Y., Zhou N., Li J., Jiang B., Cheng J., Li C. (2022). Mechanosensitive Piezo1 channels mediate renal fibrosis. J. Clin. Investig..

[56] Fu R., Wang W., Huo Y., Li L., Chen R., Lin Z., Tao Y., Peng X., Huang W., Guo C. (2024). The mechanosensitive ion channel Piezo1 contributes to podocyte cytoskeleton remodeling and development of proteinuria in lupus nephritis. Kidney Int..

[57] Baecker D. (2025). Progress and challenges in the development of clinically viable Piezo1 inhibitors. Futur. Med. Chem..

[58] Schultz A., Halabi A., Seitz F., Lemmens K., Wülfrath H.S., Lobmeyer M.T., Retlich S., Choi W., Soleymanlou N. (2025). Phase 1 trials of BI 764198, a transient receptor potential channel 6 inhibitor, in healthy volunteers and participants with kidney impairment. Expert Opin. Investig. Drugs.

